# Near-infrared operating lamp for intraoperative molecular imaging of a mediastinal tumor

**DOI:** 10.1186/s12880-016-0120-5

**Published:** 2016-02-17

**Authors:** Jane Keating, Ryan Judy, Andrew Newton, Sunil Singhal

**Affiliations:** Department of Surgery, University of Pennsylvania and Philadelphia VA Medical Center, 6 White Building, 3400 Spruce Street, Philadelphia, PA 19104 USA

**Keywords:** Intraoperative imaging, Cancer, Near-infrared, Indocyanine green, Mediastinal tumors

## Abstract

**Background:**

Near-Infrared (NIR) intraoperative molecular imaging is a new diagnostic modality utilized during cancer surgery for the identification of tumors, metastases and lymph nodes. Surgeons typically use headlamps during an operation to increase visible light; however, these light sources are not adapted to function simultaneously with NIR molecular imaging technology. Here, we design a NIR cancelling headlamp and utilize it during surgery to assess whether intraoperative molecular imaging of mediastinal tumors is possible.

**Methods:**

A NIR cancelling headlamp was designed and tested using NIR spectroscopy preoperatively. Next, a 46 year-old-female was referred to the thoracic surgery clinic for a 5.8 cm mediastinal mass noted on chest x-ray. Prior to surgery, she was given intravenous indocyanine green (ICG). Then, the prototype headlamp was used in conjunction with our intraoperative molecular imaging device. The tumor was imaged both in vivo and following resection prior to pathological examination.

**Results:**

NIR spectroscopy confirmed NIR light excitation of the unfiltered headlamp and the absence of NIR emitted light after addition of the filter. Next, in vivo imaging confirmed fluorescence of the tumor, but also demonstrated a significant amount of NIR background fluorescence emanating from the unfiltered headlamp. During imaging with the filtered headlamp, we again demonstrated a markedly fluorescent tumor but with a reduced false positive NIR signal. Final pathology was well-differentiated thymoma with negative surgical margins.

**Conclusions:**

NIR intraoperative molecular imaging using a systemic injection of intravenous ICG was successful in localizing a thymoma. Additionally, a simple design and implementation of a NIR cancelling headlamp reduces false positive NIR fluorescence.

## Background

Near-infrared (NIR) intraoperative molecular imaging is utilized for cancer surgery for the detection of many tumor types, including colorectal, ovarian and lung cancers [1–6]. The most commonly used and only FDA approved NIR tracer for cancer detection is indocyanine green (ICG). Benefits of NIR imaging include the use of non-ionizing radiation as the NIR spectrum involves low energy light ranging from 750 to 1400 nm [7].

During open surgery, surgeons frequently use over-the-bed operating room lights and headlamps to provide additional visible light to the surgical field. These lights can be problematic for intraoperative imaging because they emit NIR light in addition to white light that results in a false positive signal [8]. We developed and tested a novel headlamp system that filters out this false positive NIR signal without affecting intraoperative visibility or significantly altering the weight or utility of the headlamp. This approach allows a surgeon to perform a routine cancer operation without compromising his or her view while obtaining the optimal NIR excitation and visualization for cancer imaging.

## Methods

### NIR cancelling headlamp

To create the NIR cancelling headlamp, an UltraLite Plus Headlight System (Integra, AX1375BIF) was fitted with two sequential 25 mm diameter heat absorbing glass filters (Edmund Optics, #49-088). The IR-Cut filters were placed sequentially to allow visible spectral range light to pass while strongly blocking infrared rays from 780 nm and higher. Each filter is 3 mm thick and able to stand up to 300° Celsius. Thus, they are able to withstand the heat generated by headlamps over the complete duration of a surgery. Together the filters weigh approximately 70 g, so they add negligible weight to the headlamp. All construction was performed by the Research Instrumentation Shop at the Perelman School of Medicine at the University of Pennsylvania.

### Spectroscopy

The headlamp without any filtration was first tested using an Ocean Optics spectrometer (QE65 Pro). This spectrometer consists of a full frame transfer charge coupled device (FFT-CCD) image sensor that can detect signal from 200 to 1100 nm. Headlamp emission was measured by the spectrometer both with and without the filter for comparison. The conditions under which spectroscopy was performed were kept identical before and after removal of the filter. The spectrometer was held as fixed and close to the illuminated headlamp as possible without touching the surface. Additionally the level of ambient light was held constant throughout the entirety of the experiment.

### Patient

**Fig. 1:**
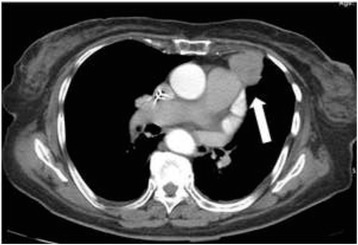
Preoperative CT scan of the chest. Incidentally discovered 5.8 cm anterior mediastinal mass noted on preoperative CT scan. The arrow points to the tumor

A 46 year-old-female was enrolled for intraoperative imaging in a thoracic surgery clinic for an incidentally discovered mediastinal mass on chest x-ray. The mass was noted to be 5.8 cm on preoperative CT scan (Fig. 1). Following informed and written consent, the patient was enrolled in a clinical trial studying NIR intraoperative molecular imaging of mediastinal tumors. The clinical trial protocol was approved by the University of Pennsylvania Institutional Review Board.

Twenty-four hours prior to surgery, the patient underwent intravenous injection of 5 mg/kg of indocyanine green (ICG) via antecubital vein.

### Reagent

Indocyanine green (ICG) (Akorn, Lake Forest, IL and Pulsion, Feldkirchen, Germany) is a water-soluble anionic, amphiphilic NIR fluorophore with an excitation wavelength of 790 nm and an emission wavelength of 830 nm and a molecular weight of 774.9 kDA.

### Imaging device

Macroscopic surgical fluorescent imaging was performed using the Iridium (Visionsense, New York, NY) which is capable of emitting and absorbing light in the NIR spectrum. The Iridium is a high definition (HD) 3D camera (λex 790 nm and λem 830 nm). Positive and negative controls were used for all images. The imaging device was held 10 in. above the specimen for each measurement for consistency of focal length.

### Tumor-to-background ratio

We used region-of-interest software and HeatMap plugin within ImageJ (http://rsb.info.nih.gov/ij/; public domain free software developed by National Institutes of Health) in order to quantitate tissue fluorescence. The NIR fluorescence of the tumor and surrounding normal mediastinal subcutaneous fat in vivo were measured in order to calculate a mean tumor-to-background ratio (TBR). Four total measurements of both tumor and background fluorescence were recorded. The first measurement was taken at the approximated center of the tumor. The following three measurements were taken 1 cm from the centimeter of the tumor in three radial directions (12 o’clock, 4 o’clock and 8 o’clock). These four measurements were used to calculate mean tumor fluorescence. Similarly, background fluorescence was measured from surrounding subcutaneous mediastinal fat in four radial directions extending from the center of the tumor (superior, inferior, medial and lateral). The mean background fluorescence was then calculated, and mean fluorescence from both the tumor and surrounding background were used to generate a mean TBR. An identical technique was used for *ex vivo* TBR measurement. For *ex vivo* imaging, the background fluorescence was taken from a non-reflective blue cloth background.

## Results

### NIR spectroscopy of filtered headlamp detects deleted NIR emission

**Fig. 2:**
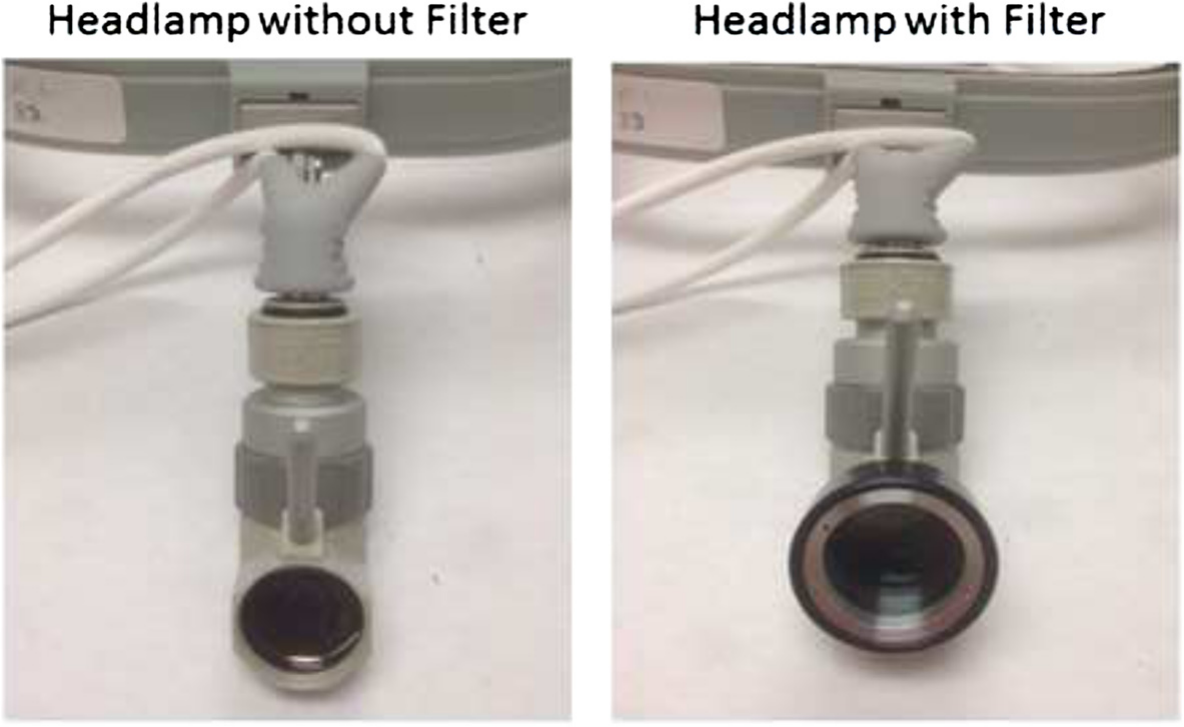
Headlamp with and without NIR cancelling filter. Typical surgical headlamp with and without the addition of a removable filter that functions to cancel NIR light emitted from the headlamp onto the surgical field

**Fig. 3:**
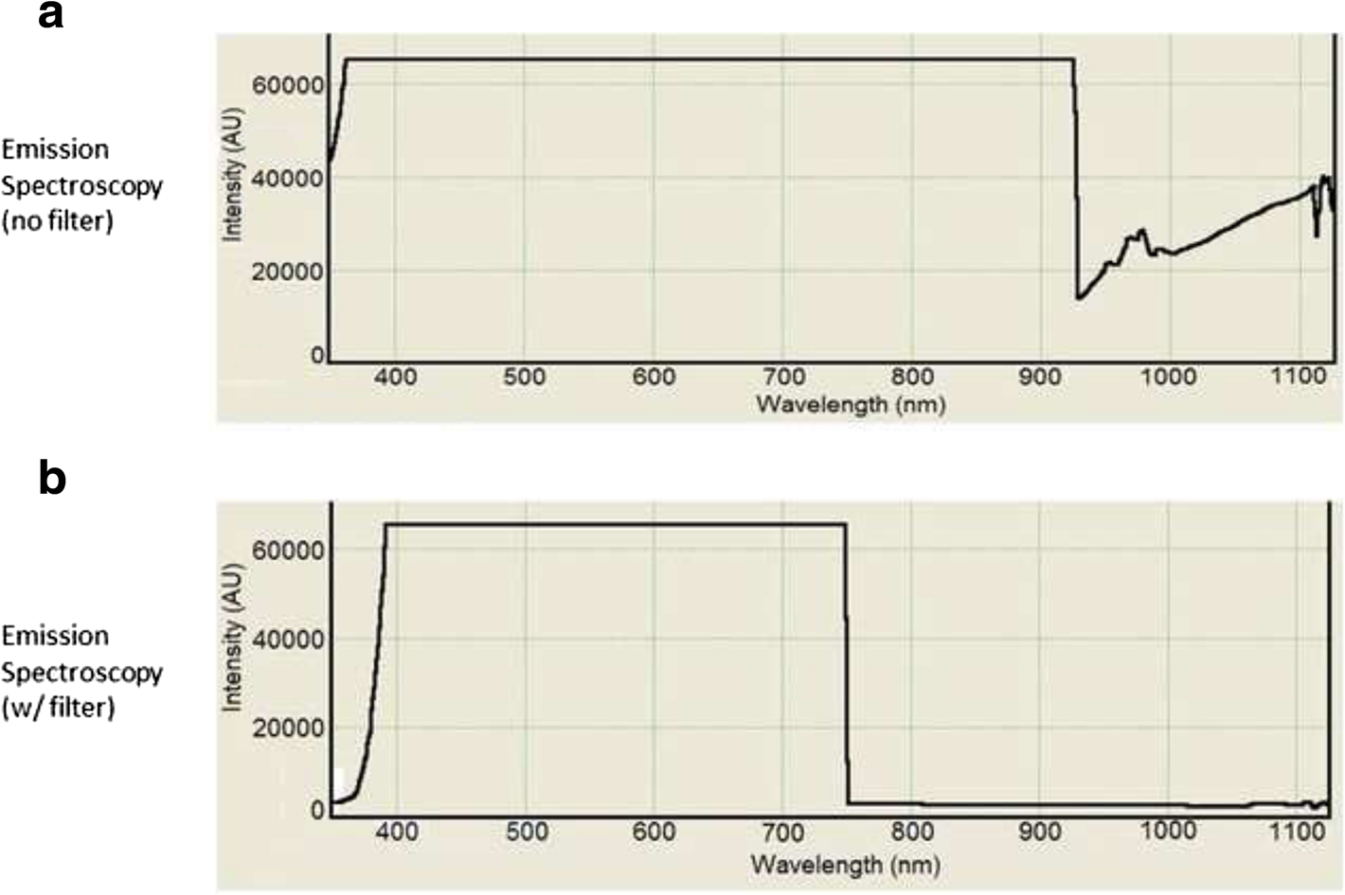
NIR Spectroscopy. **a** Spectroscopy demonstrating NIR light (780-950 nm) emitted from the surgical headlamp prior to addition of the filter. **b** Spectroscopy shows absence of light greater than 780 nm after addition of the filter to the headlamp

The headlamp was configured in order to allow ease of filter application and removal (Fig. 2). First, headlamp emission without any filtration was measured by the spectrometer, which confirmed light excitation greater than 780 nm (Fig. 3a) that falls within the NIR range. This falls within the detectable range of our imaging device and causes false positive fluorescence during imaging. With the addition of the NIR cancelling filter, spectroscopy was repeated, which showed no light emitted above 780 nm within the NIR spectrum (Fig. 3b).

### Filtered headlamp reduces in vivo false positive NIR fluorescence

Twenty-four hours after ICG injection, the patient was brought to the operating room. Following standard anesthesia, intubation and sternotomy, the tumor was localized, the overhead lights were dimmed, and our NIR imaging device was draped and suspended above the patient. At this time, the surgeon was wearing the standard unfiltered headlamp*.* In vivo imaging confirmed fluorescence of the tumor but also demonstrated a significant amount of NIR background fluorescence emanating from the headlamp. The surgeon’s headlamp was replaced with the modified prototype headlamp and the surgical bed was re-imaged.

**Fig. 4:**
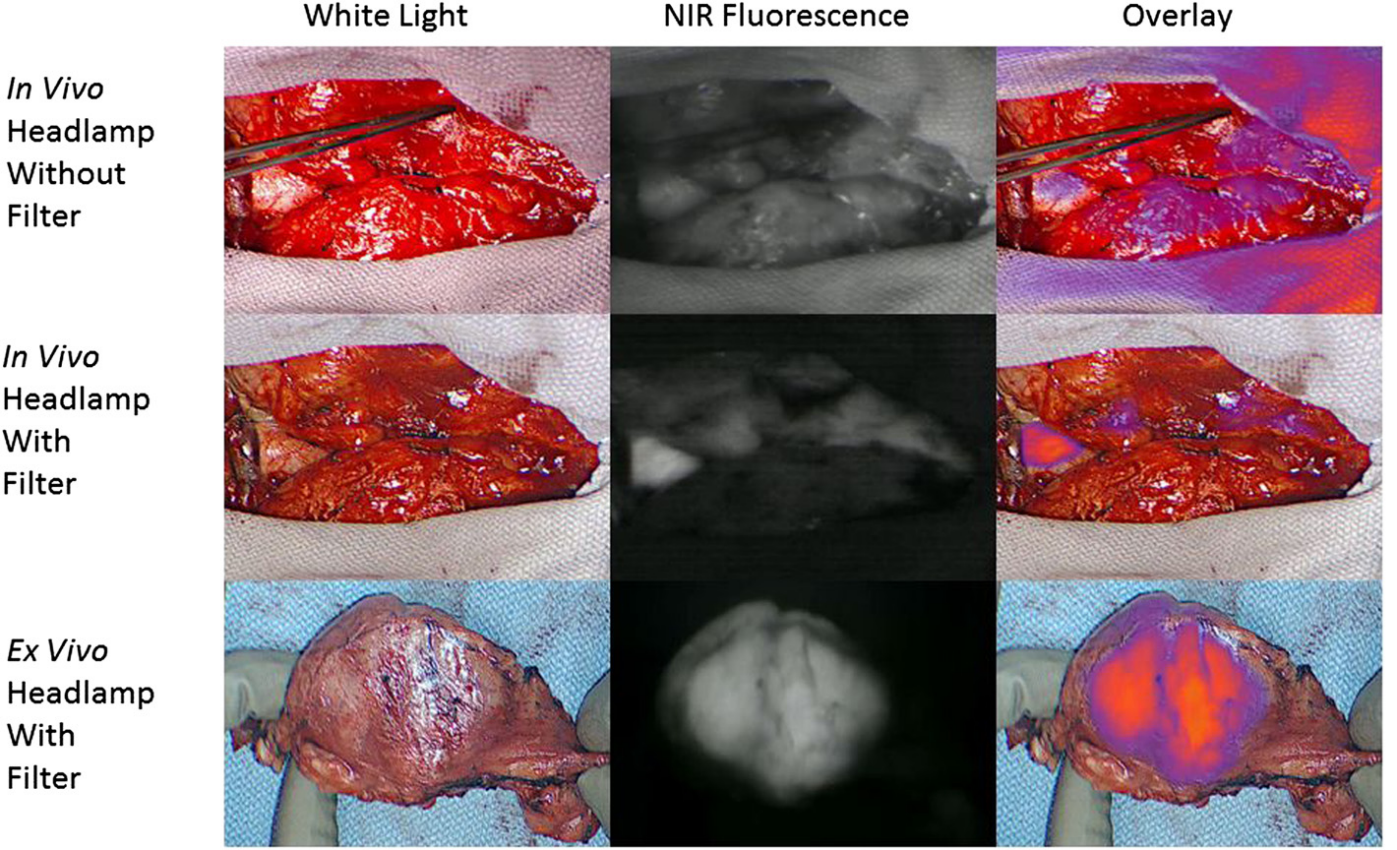
NIR Imaging of mediastinal mass in vivo and *ex vivo.* First row: In vivo NIR intraoperative molecular imaging with the use of a typical surgical headlamp. White light, NIR fluorescence and overlay images are shown. Note the false positive NIR fluorescence detected with our intraoperative imaging device when the headlamp is used without a filter. Second row: The addition of the NIR cancelling filter removes the NIR signal emitted from the headlamp and thus reduces false positive fluorescence. Note the tumor is markedly fluorescent in vivo. Third row: With the filtered headlamp still in place, the fluorescent tumor is reimaged *ex vivo*. Pathology demonstrated thymoma with negative surgical margins

Both in vivo and *ex vivo* imaging of the mediastinal mass again demonstrated a markedly fluorescent tumor without the additional NIR fluorescence from the headlamp (Fig. 4). Mean in vivo tumor fluorescence without headlamp filtration was noted to be 104.3 A.U. (+/- 4.0) while background fluorescence was 63.7 A.U. (+/- 1.7). The mean TBR was 1.6. Mean in vivo tumor fluorescence with headlamp filtration was 107.0 A.U. (+/- 3.6), mean background fluorescence was reduced to 35.4 A.U. (+/- 2.0), and mean TBR was 3.0. In other words, with the addition of the filtered headlamp, there was little difference between tumor fluorescence, however background fluorescence was significantly decreased which allowed for increased TBR. During *ex vivo* imaging with the filtered headlamp still in place, mean tumor fluorescence was 116.5 A.U. (+/- 6.4), mean background fluorescence was 30.3 A.U. (+/- 1.7), and mean TBR was 3.8. Final pathology of the mediastinal tumor demonstrated thymoma with negative margins.

## Discussion

NIR intraoperative molecular imaging using systemic ICG successfully located a mediastinal tumor. Intraoperative molecular imaging is a burgeoning area of technology and research that has promising applications for surgical oncology. With its increasing popularity, operating rooms around the country are being built specifically with the capability of convenient housing of intraoperative imaging devices [9–11]. However, troubleshooting is still necessary in many instances. One area of improvement is the reduction of false-positive NIR signal from operating room lights and headlamps.

Here we describe the successful implementation of a prototype headlamp for the removal of NIR signal from a standard surgical headlamp. We first confirm preoperatively NIR subtraction using spectroscopy and then show its utility in the operating room during intraoperative molecular imaging of a mediastinal mass.

As NIR intraoperative molecular imaging is becoming increasingly utilized, simple adjustments like the one we have described are necessary for the successful and practical implementation of this imaging technology. Additionally, in the future it is likely that the producers of surgical lights and headlamps will be encouraged to permanently remove the NIR signal emitted from standard operating room devices as this signal provides no additional visibility to the surgeon but instead causes distracting false positive signal during these procedures. Based on our results with NIR intraoperative molecular imaging on a series of patients, a larger clinical trial of NIR intraoperative molecular imaging for mediastinal tumors is underway.

## Conclusions

The addition of NIR-cancelling glass filters to a surgical headlamp decreased background fluorescence during intraoperative molecular imaging of a thymoma. This simple modification may improve localization and intraoperative margin assessment during open oncology surgery utilizing intraoperative molecular imaging.

### Ethics approval and consent to participate

This clinical trial protocol was approved by the University of Pennsylvania Institutional Review Board. Written informed consent was obtained from the patient for participation in the trial.

### Consent for publication

Written informed consent was obtained from the patient for publication of this manuscript and any accompanying images. A copy of the written consent is available for review by the Editor-in-Chief of this journal.

## Abbreviations

A.U.: arbitrary units
ICG: indocyanine green
NIR: near-infrared
TBR: tumor-to-background ratio
